# Pyridoxic Acid as a Novel Endogenous Biomarker for OAT-Mediated Clearance of Meropenem: A Population Pharmacokinetic Modeling and PK/PD-Response Analysis in Patients with Sepsis

**DOI:** 10.3390/pharmaceutics18091113

**Published:** 2026-09-04

**Authors:** Zihan Lei, Hao Liang, Qin Cheng, Weijie Kong, Yipeng Du, Xueting Yao, Feifei Feng, Yuyan Jin, Wenting Wang, Haiyan Li, Ming Lu, Dongyang Liu, Ning Shen

**Affiliations:** 1 Department of Pulmonary and Critical Care Medicine, Peking University Third Hospital, Beijing 100191, China; 2 Drug Clinical Trial Center, Peking University Third Hospital, Beijing 100191, China; 3 Department of Clinical Pharmacology, Jiangsu Hengrui Pharmaceuticals Co., Ltd., Shanghai 200135, China; 4 Center of Clinical Medical Research, Institute of Medical Innovation and Research, Peking University Third Hospital, Beijing 100191, China; 5 Center of Infectious Disease, Peking University Third Hospital, Beijing 100191, China

**Keywords:** translational biomarker, precision medicine, pyridoxic acid, OAT transporters, pharmacokinetic/pharmacodynamic, sepsis, meropenem

## Abstract

**Background/Objectives:** Meropenem pharmacokinetic variability in sepsis often leads to suboptimal exposure and therapeutic failure. Existing covariates like creatinine clearance (CLcr) only partially explain this variability. This study evaluated pyridoxic acid (PDA), an endogenous biomarker of OAT1/3 transporters, as a novel covariate to quantify active tubular secretion and explore pharmacokinetic/pharmacodynamic (PK/PD) linkages with clinical outcomes. **Methods:** A population PK (PopPK) model was constructed using data from a prospective septic cohort (*n* = 28). Subsequent exposure-response analysis was conducted in an expanded cohort (*n* = 49), and Monte Carlo simulations were utilized to evaluate various dosing regimens. **Results:** The PopPK analysis suggested that PDA may complement CLcr in characterizing meropenem clearance variability. While CLcr explained 10.7% of inter-individual variability (IIV) in clearance, the inclusion of PDA explained an additional 13.7%, reducing total IIV from 50.8% to 26.4%. Achieving a stringent target of 100%fT > 4MIC was significantly associated with a rapid decline in procalcitonin (*p* = 0.027), establishing a key PD endpoint. Simulations demonstrated that standard dosing (1 g q8h, 1 h infusion) is insufficient for patients with normal or augmented renal function. Target attainment was highly dependent on PDA levels. **Conclusions:** PDA is a valuable translational biomarker for OAT-mediated clearance. To achieve 100%fT > 4MIC, we recommend (i) 1 g q8h with 3 h infusion for patients with low CLcr and high PDA levels (MIC = 0.5 mg/L), and (ii) an intensified regimen of 2 g q8h with 3 h infusion for patients with normal CLcr and low PDA levels or high resistance risk (MIC ≥ 2 mg/L).

## 1. Introduction

Meropenem is a frontline broad-spectrum carbapenem for severe sepsis [1,2]. As a time-dependent antibiotic, its bactericidal activity is optimally correlated with the percentage of time that the free drug concentration remains above the minimum inhibitory concentration (%fT > MIC) [3,4].

However, in critically ill patients, profound pathophysiological changes lead to highly variable pharmacokinetics (PK) and unpredictable exposure [5]. Pathophysiological alterations such as altered volume of distribution, hypoalbuminemia, hyperdynamic states, and organ dysfunction can significantly affect drug clearance (CL) and exposure [6,7]. Renal function is a major determinant of meropenem elimination, as the drug is primarily excreted unchanged via the kidneys through both glomerular filtration and active tubular secretion mediated by organic anion transporters (OAT1 and OAT3) [8,9].

Currently, creatinine clearance (CLcr) alone fails to capture the full spectrum of renal clearance, frequently leading to suboptimal dosing [10,11,12,13]. Recently, 4-pyridoxic acid (PDA), a major metabolite of vitamin B6, has emerged as a promising endogenous biomarker for OAT function. Initially identified through comprehensive metabolomics screening in cynomolgus monkeys, the utility of PDA as a sensitive endogenous probe for OAT activity has been subsequently validated in clinical trials involving healthy human volunteers [14,15,16]. Despite these findings, the clinical applicability of PDA in predicting the pharmacokinetics of OAT-substrate antibiotics, such as meropenem, specifically in critically ill patients with sepsis remains unexplored.

Furthermore, while achieving a PK/PD target such as %fT > MIC is a recognized surrogate for efficacy [2,17], its linkage to direct clinical outcomes in a heterogeneous sepsis population requires further validation [18,19]. This necessitates comprehensive exposure-response (ER) analysis incorporating endpoints such as 28-day mortality, clinical cure, and inflammatory biomarker dynamics [20,21]. Establishing which specific PK/PD target (e.g., 100%fT > MIC) is most strongly associated with clinical success is a critical prerequisite for meaningful dose optimization.

Therefore, this study aims to (i) evaluate whether integrating PDA as a mechanistic covariate improves meropenem PopPK model precision compared to CLcr alone; (ii) define the specific pharmacodynamic index most strongly associated with clinical outcomes; and (iii) utilize Monte Carlo simulations to develop optimized, model-informed dosing regimens that achieve rigorous target attainment across diverse renal profiles.

## 2. Methods

### 2.1. Study Design and Patient Population

This single-center, observational study was conducted between January 2022 and December 2024. Adult patients (≥18 years) diagnosed with sepsis or septic shock according to the Sepsis-3 criteria and receiving intravenous meropenem were eligible for inclusion. Exclusion criteria were as follows: pregnancy or breastfeeding; administration of probenecid, gentamicin, or streptomycin within the previous 14 days; or intake of vitamin B6 within the previous 14 days. Patients were categorized into normal renal function, acute kidney injury (AKI), and continuous renal replacement therapy (CRRT) groups based on the Kidney Disease: Improving Global Outcomes (KDIGO) criteria at baseline. This study was reviewed and approved by the Peking University Third Hospital Institutional Review Board. Informed consent was obtained from all individual participants (or their legal guardians) included in the study.

The overall analytical framework comprised two levels of cohorts. For the development of the PopPK model, a subset of patients who underwent intensive PK sampling was utilized. This subset was selected based on the feasibility of intensive sampling (e.g., presence of dedicated central venous access and availability of research staff). Subsequently, individual meropenem exposures for all patients in the full cohort were calculated through simulations based on the final PopPK model incorporating their specific patient-level covariates. The full cohort was then included in the exposure-response (PK/PD) analysis to evaluate clinical outcomes and biomarker dynamics.

### 2.2. Blood Sampling and Meropenem Assay

Intensive blood sampling was performed to characterize the meropenem pharmacokinetic profile. Sample were collected at five key time points: pre-dose (trough), immediately after the end of infusion (peak), at 0.5 h post-infusion, at 3.5 h post-infusion for the q8h regimen (or 5.5 h for the q12h regimen), and immediately before the next dose (0 h pre-next dose). All collected samples were analyzed for meropenem concentrations, while the PDA concentration was measured exclusively in the initial pre-dose (baseline) sample.

Meropenem and PDA concentrations in plasma were quantified using a validated liquid chromatography-tandem mass spectrometry (LC-MS/MS) method. Tolbutamide (Darmstadt, Germany) and PDA-d3 (Toronto, ON, Canada) were used as the internal standards (IS) for meropenem (Dalian, China) and PDA (Toronto, ON, Canada), respectively. Chromatographic separation was performed using a Waters (Milford, MA, USA) XBridge C18 column for meropenem and a Waters HSS T3 column for PDA. The calibration curves were linear over the ranges of 50.0–5000 ng/mL for meropenem and 0.125–16.0 ng/mL for PDA. All serum samples were immediately centrifuged and stored at −80 °C until analysis. Stability tests demonstrated that both meropenem and PDA were stable when stored at −80 °C for up to 2 months. Detailed bioanalytical conditions and validation parameters, including precision and accuracy, are summarized in Supplementary Table S1.

### 2.3. Population Pharmacokinetic Modeling

A PopPK model was developed using nonlinear mixed-effects modeling (NONMEM software, version 7.2.0; Icon Development Solutions, Dublin, Ireland). The first-order conditional estimation method with interaction (FOCE-I) was employed. Base model development began with one- and two-compartment structural models with zero-order input and first-order elimination. IIV was assessed using an exponential error model, and residual variability was evaluated using combined error models.

An exploratory univariate analysis was conducted to identify potential covariates for inclusion. The individual empirical Bayesian estimates of the ETA on key PK parameters (ETA-CL and ETA-VC) from the final base model were extracted. Using R software (version 4.4.1), the correlations between these ETA values and continuous clinical covariates—including CLcr (estimated by both the Cockcroft–Gault and MDRD equations), body weight, 24 h urine output, PDA level, procalcitonin (PCT), white blood cell (WBC), and other laboratory test indicators—were assessed by calculating Pearson or Spearman correlation coefficients based on the data distribution. The relationship of categorical covariates, namely sex and renal-status (nonAKI, AKI, CRRT), to the ETAs was compared using non-parametric tests.

Following the exploratory analysis, covariates that demonstrated an absolute correlation coefficient of |*r*| > 0.3 or a statistically significant association (*p* < 0.05) were incorporated into a formal stepwise covariate modeling procedure in NONMEM. This procedure consisted of forward inclusion (ΔOFV ≥ 3.84; *p* < 0.05, 1 degree of freedom) followed by backward elimination (ΔOFV ≥ 6.63; *p* < 0.01, 1 degree of freedom) to establish the final parsimonious model. Covariates providing substantial clinical and mechanistic rationale, supported by significant variance reduction and visual predictive improvements, could be retained in the final model as an exception to the standard backward elimination criteria.

### 2.4. Model Evaluation and Validation Methodology 

Model performance was assessed using goodness-of-fit plots, visual predictive checks (VPC), and Sampling Importance Resampling (SIR). The final model was validated internally, and parameter uncertainty was characterized using the SIR method.

### 2.5. PK-PD-Endpoint Analysis

Univariable analyses were performed to assess the associations between each PK/PD exposure target and the clinical/laboratory outcomes by R software (version 4.4.1). The following PK/PD exposure targets were evaluated: Continuous Targets: %fT > MIC, %fT > 4MIC, trough concentration (C_min_) and maximum plasma concentration (C_max_); Binary Targets: achievement of various thresholds (50%fT > MIC, 100%fT > MIC, 50%fT > 4MIC, 100%fT > 4MIC). The MIC was based on microbiological testing when available; otherwise, the institutional epidemiologic MIC value for the suspected pathogen was applied. Meropenem exposure was simulated based on the final PopPK model.

The primary outcomes were all-cause mortality and 28-day mortality. Secondary outcomes included in-hospital mortality, clinical failure (defined as a composite of persistence of signs/symptoms, change of antibiotic, or death), clinical failure at day 10, ICU length of stay, duration of mechanical ventilation during ICU stay, and changes in laboratory biomarkers (WBC, PLT and PCT) after 5 days of meropenem therapy [21]. Binary outcomes were analyzed using univariable logistic regression. For continuous outcomes that met the normality assumption, linear regression was used. For continuous outcomes that did not, the Mann–Whitney U test and Spearman’s rank correlation were applied. For the primary analysis of PCT reduction, given the non-normality of the model residuals, we supplemented the primary non-parametric comparison with bootstrap resampling (2000 replicates) using the bias-corrected and accelerated (BCa) method to obtain robust 95% confidence intervals. This approach was applied to both unadjusted and propensity-score-adjusted linear regression models, with the propensity score derived from the SOFA score. A two-sided *p*-value < 0.05 was considered statistically significant.

### 2.6. Model-Based Simulations and Dosing Regimen Optimization Framework

The Monte Carlo simulation was performed using the final PopPK model. The primary targets for bactericidal efficacy were set at 100%fT > MIC and 100%fT > 4MIC. Neurotoxicity was defined as a trough concentration exceeding 64.2 mg/L [22]. This aggressive target of 100%fT > 4MIC was selected based on emerging evidence that higher exposure may be required in critically ill patients to ensure optimal clinical outcomes and suppress resistance emergence, a choice further supported by the exposure-response trends identified in this study.

A comprehensive set of clinically relevant regimens was simulated to explore the impact of dose, dosing interval, and infusion duration: 1 g q8h (1 h infusion), 1 g q12h (1 h infusion), 1 g q8h (3 h infusion), 1 g q12h (3 h infusion), 1 g q12h (6 h infusion), and 2 g q8h (3 h infusion). For each regimen, 5000 subject profiles were simulated, incorporating the parameter variability and covariate distributions from the final PopPK model. The PTA was calculated across a MIC range of 0.125 to 32 mg/L. A PTA threshold of 90% was employed as the primary criterion for recommending optimized dosing regimens. The highest C_min_ occurring in patients with impaired renal function was simulated to ensure it did not exceed the neurotoxicity threshold of 64.2 mg/L.

### 2.7. Statistical Analysis

All statistical analyses were performed using R software (version 4.4.1). Continuous variables are presented as mean ± standard deviation or median (interquartile range), and categorical variables as frequencies (percentages). For comparisons between groups, normally distributed continuous variables were analyzed using Student’s *t*-test or one-way analysis of variance (ANOVA), while non-normally distributed variables were compared using the Mann–Whitney U test or Kruskal–Wallis test. Categorical variables were compared using the Chi-square test or Fisher’s exact test. As detailed in the PK/PD-Endpoint Analysis section, logistic, linear regression and non-parametric methods were utilized to evaluate the exposure-response relationships. Missing data for continuous variables were handled using median imputation to preserve sample size and statistical power. All statistical tests were two-sided, and a *p* value < 0.05 was considered statistically significant.

## 3. Result

### 3.1. Patient Characteristics and Meropenem Concentrations

**Figure 1:**
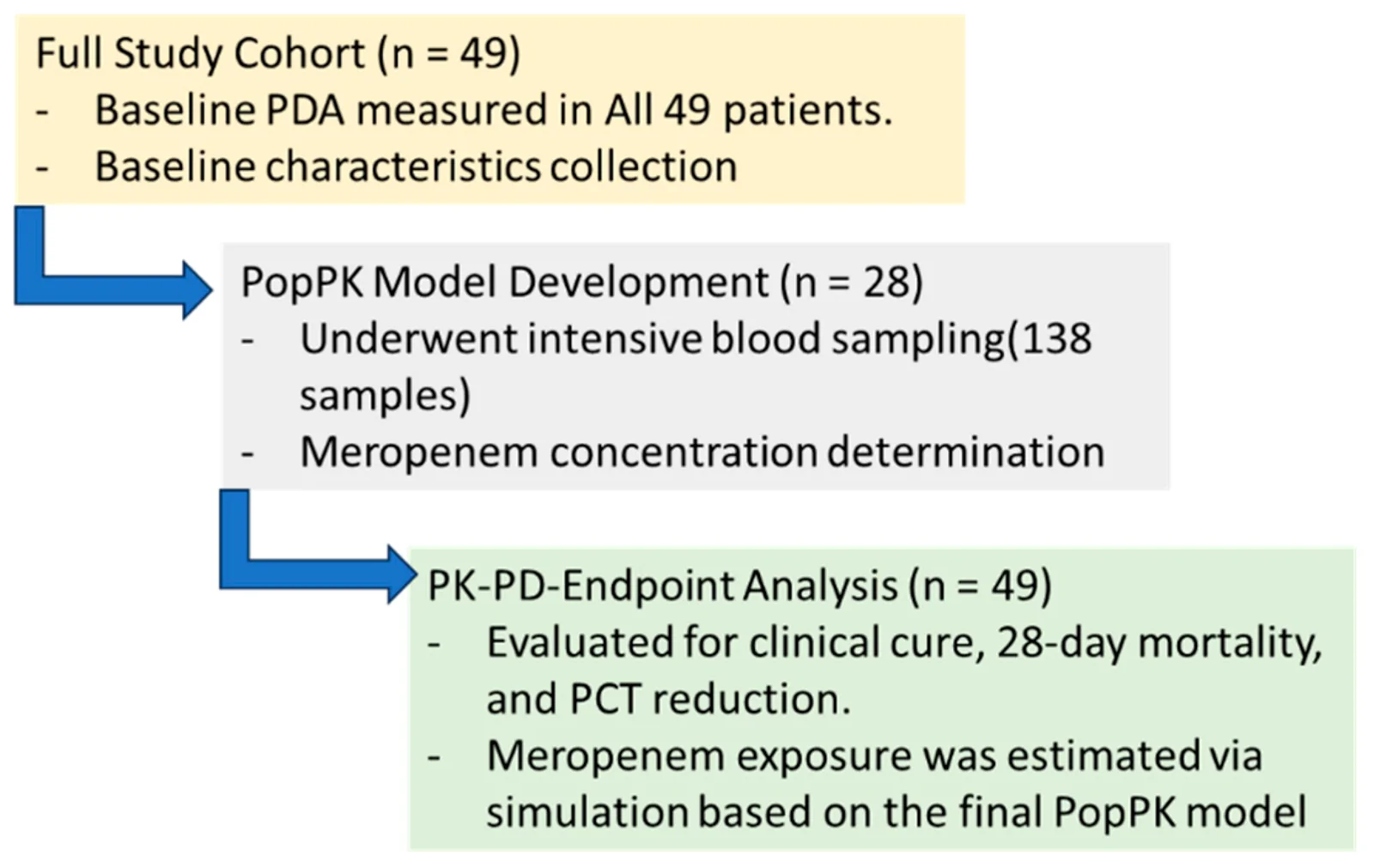
Study Cohort design and analytical framework.

**Figure 2:**
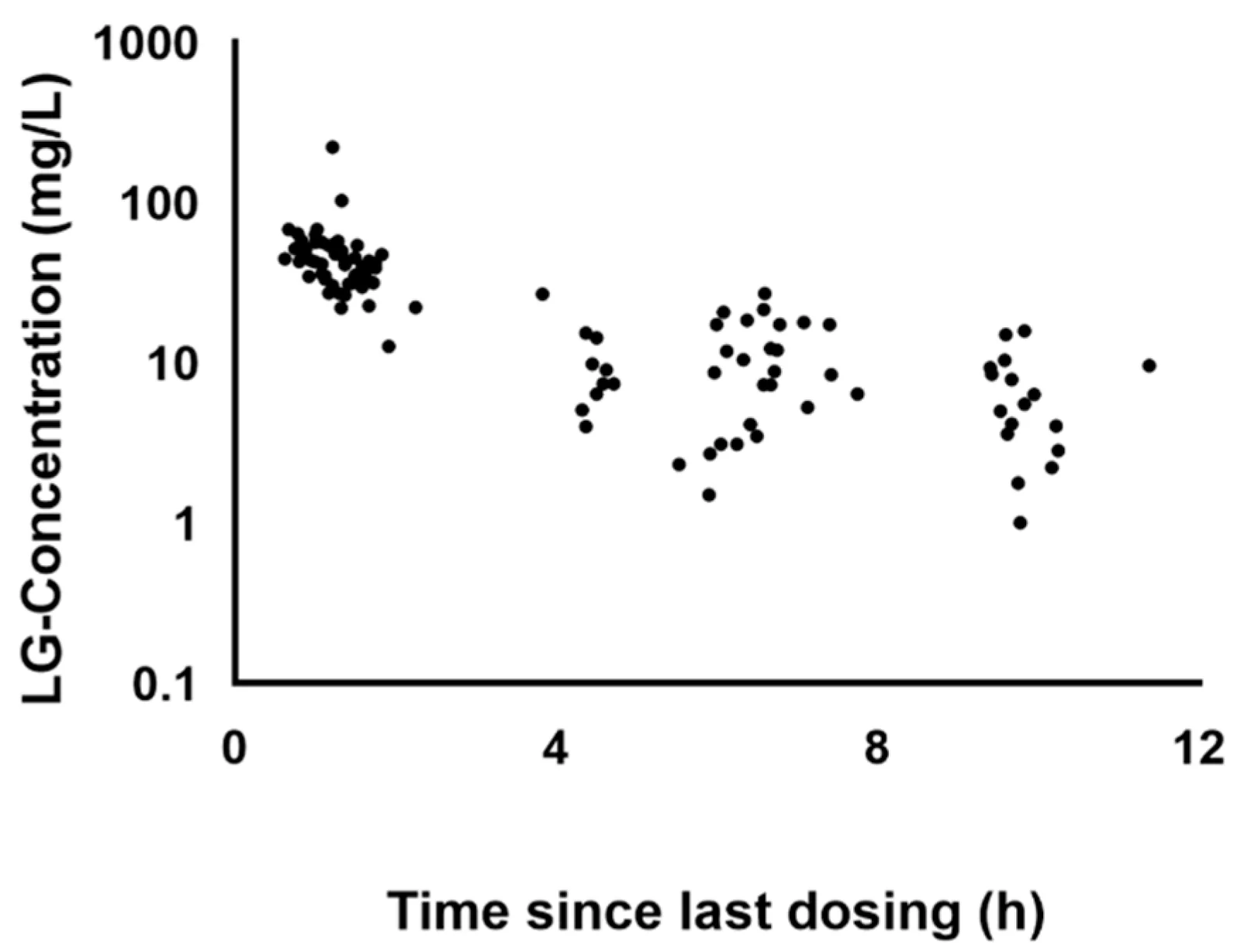
Scatter plot of meropenem drug concentration versus time since the last dose from 28 patients.

The relationship between the full study cohort (*n* = 49) and the pharmacokinetic (PK) subset (*n* = 28) is illustrated in Figure 1. A total of 49 patients with sepsis were enrolled in this study for the exposure-response (ER) analysis. From this cohort, a subset of 28 patients underwent intensive pharmacokinetic sampling, yielding 138 blood samples for PopPK model development. Based on baseline renal function, the full cohort was categorized into normal renal function (*n* = 21), AKI without CRRT (*n* = 21), and CRRT (*n* = 7) groups. Baseline PDA concentrations were universally measured in all 49 enrolled patients. The baseline demographic and clinical characteristics are summarized in Table 1. The measured meropenem serum concentrations exhibited substantial variability, ranging from 0.28 to 223.12 mg/L, which was considered sufficient for population model development (Figure 2).

During the study period, the meropenem dosage regimens varied among patients. Specifically, 3 patients received a regimen of 0.5 g q12h, 19 patients received a regimen of 1.0 g q8h and 27 patients received a regimen of 1.0 g q12h. All doses were administered via intravenous infusion over 1 h.

### 3.2. Population Pharmacokinetic Model Development

#### 3.2.1. Base Model

The pharmacokinetics of meropenem were best described by a two-compartment structural model with first-order elimination. The base model, which included IIV on CL and the volume of the central compartment (Vc), provided a robust foundation for subsequent covariate analysis.

(1) $$\frac{dA_{C}}{dt}=-CL\times \frac{A_{C}}{V_{C}}-Q\times \left(\frac{A_{C}}{V_{C}}-\frac{A_{p}}{V_{p}}\right),A_{C}(0)=0$$

(2) $$\frac{dA_{p}}{dt}=Q\times \left(\frac{A_{C}}{V_{C}}-\frac{A_{p}}{V_{p}}\right),A_{p}(0)=0$$

Base model equation: Ac and Ap denote the drug amount in the central compartment and peripheral compartment. Vc and Vp denote the distribution volume of the central compartment and peripheral compartments. Q are the clearance between the central and peripheral compartments. CL is the systemic clearance.

#### 3.2.2. Covariate Model (Model 1)

The detailed, step-by-step log of the automated SCM procedure, including all OFV changes and *p*-values, is provided in Supplementary Table S2. In the stepwise covariate screening, CLcr (estimated by Cockcroft-Gault equation) was identified as a significant covariate on meropenem clearance. Additionally, serum albumin (ALB) and the pre-defined GROUP (renal function classification) were significant covariates on Vc (Equation (4)). These relationships were incorporated into the model using power functions, establishing Model 1.

#### 3.2.3. Final Covariate Model (Model 2) with Pyridoxic Acid (PDA)

Exploratory analysis revealed a piecewise linear relationship between PDA levels and meropenem clearance. The PDA cutoff value was estimated as a structural parameter within the PopPK model, identified at 26.5 ng/mL. Below this threshold, no correlation with clearance was observed; above this threshold—indicative of impaired OAT function—a significant linear correlation with decreased meropenem clearance was evident.

**Figure 3:**
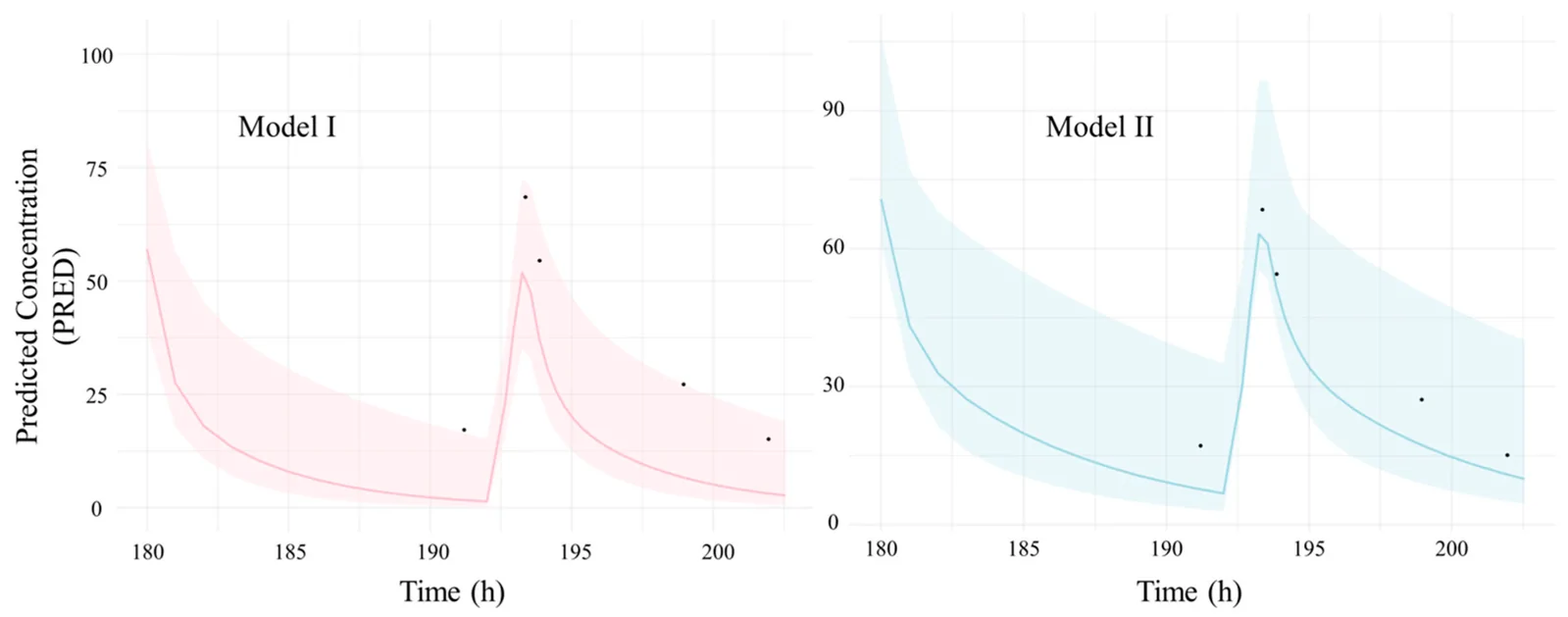
Examples of individual drug concentration-time profiles simulated by the model before (model 1) and after the inclusion of PDA (model 2): patients 1021 who have high PDA level and renal Impairment. Points: Observed values; Lines: Predicted values.

In accordance with our predefined composite criteria, incorporating PDA during forward inclusion resulted in a statistically significant OFV reduction (5.672, *p* < 0.05); however, it did not reach statistical significance during backward elimination. Exploratory analysis revealed a piecewise linear relationship between PDA and meropenem clearance. Furthermore, retaining PDA reduced the inter-individual variance of clearance, translating to a substantial decrease in the coefficient of variation from 40.1% in Model 1 to 26.4% in Model 2. The combined model incorporating both CLcr and PDA demonstrated superior predictive performance, particularly in septic patients with severe renal impairment (low CLcr and elevated PDA), as illustrated in Figure 3. While maintaining comparable predictive accuracy across the broader population, this specific subgroup improvement was further corroborated by stratified visual predictive checks (Supplementary Figure S2). Therefore, because PDA is mechanistically supported by OAT-mediated tubular secretion and provides these substantial predictive improvements, it was retained in the final model as a piecewise linear function on clearance (Model 2, Equation (3)).

(3) $$CL\left(L/h\right)=\left\{\begin{array}{c} 7.21\times {\left(\frac{CLcr}{2.62}\right)}^{0.29}-0.042\times 26.5;\;PDA\leq 26.5\;ng/mL\\ 7.21\times {\left(\frac{CLcr}{2.62}\right)}^{0.29}-0.042\times PDA;\;PDA>26.5\;ng/mL \end{array}\right\}$$

(4) $$Vc\left(L\right)=\left\{\begin{array}{c} 7.41\times {\left(\frac{ALB}{31.4}\right)}^{-3.67};\;sepsis\;with\;normal\;renal\;function\\ 7.41\times {\left(\frac{ALB}{31.4}\right)}^{-3.67}\times 2.02;sepsis\;with\;acute\;kidney\;injury\\ 7.41\times {\left(\frac{ALB}{31.4}\right)}^{-3.67}\times 4.08;\;sepsis\;with\;CRRT \end{array}\;\right\}$$

In Equations (3) and (4), the constants 2.62 L/h and 31.4 g/L correspond to the median values of CLcr and ALB observed in the PopPK study population, respectively.

The final parameter estimates for Model 2 are summarized in Table 2. The typical population values were estimated as 7.41 L for Vc and 7.21 L/h for CL. The inclusion of CLcr, PDA, ALB, and GROUP as covariates successfully accounted for a substantial portion of the observed inter-individual variability, reducing IIV for both Vc (0.069) and CL (0.052).

### 3.3. Model Evaluation and Validation Results

The goodness-of-fit plots for the final covariate model are presented in Supplementary Figure S1. The diagnostic plots demonstrate a satisfactory description of the data by the proposed model. Visual inspection reveals that the observed concentrations were in close agreement with both the population predicted (PRED) and individual predicted (IPRED) concentrations, with data points randomly and symmetrically distributed around the line of identity. Notably, the IPRED plot shows a tighter correlation compared to the PRED plot, indicating that the inclusion of inter-individual variability appropriately captures the individual PK profiles. Furthermore, the conditional weighted residuals (CWRES) plots show no systematic bias. The residuals are randomly scattered around the zero line across the range of population predictions and time intervals, with the majority of values falling within the range of −2 to 2, confirming the absence of significant model misspecification and supporting the adequacy of the residual error model.

**Figure 4:**
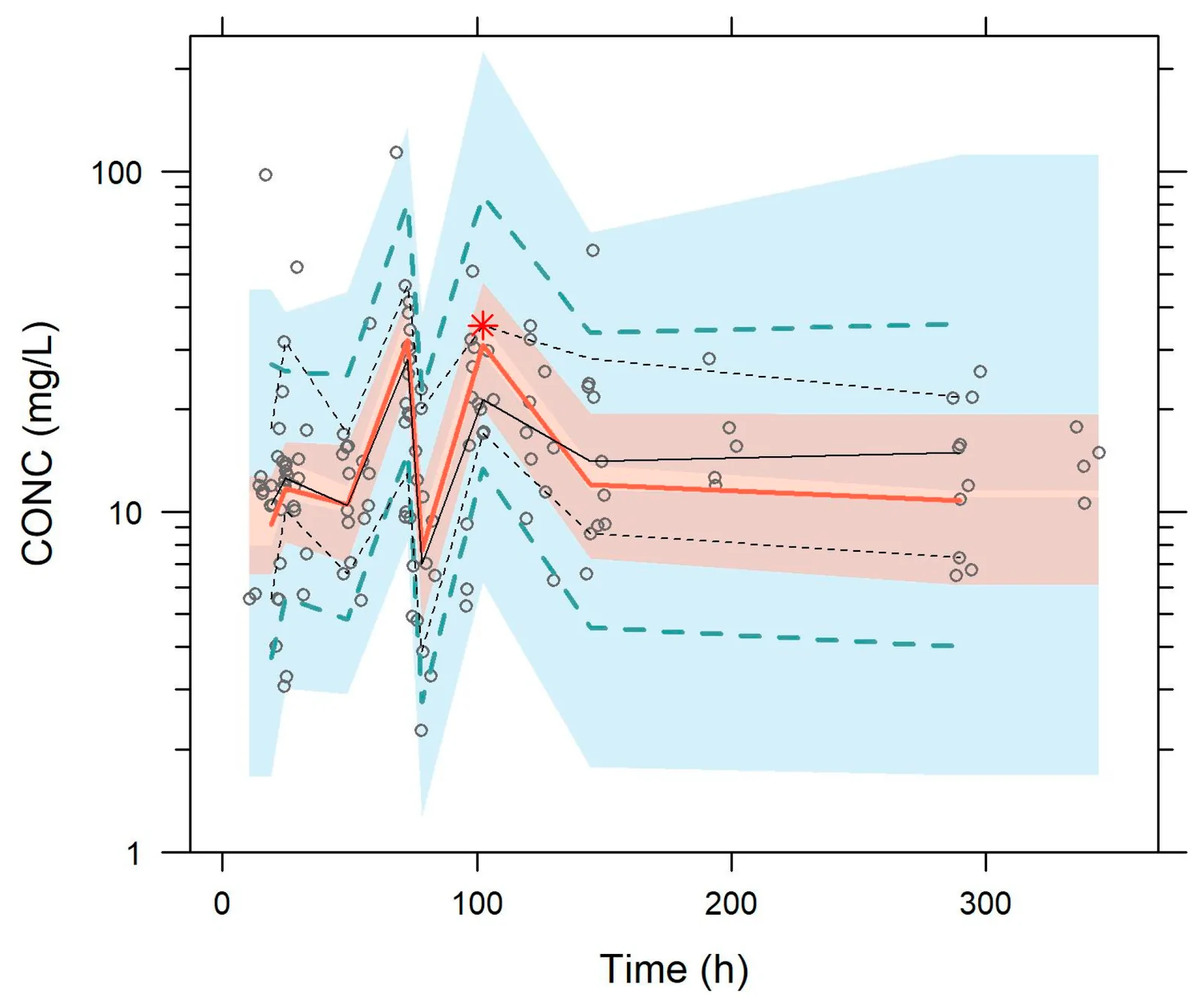
Prediction-corrected visual predictive check (pcVPC) of the meropenem final population pharmacokinetic model. The black circle represents observed PK data. The black and red solid line represents the 50th percentile for the observed and simulated data, respectively (after prediction correction). The black dotted line lines represent the 5th and 95th percentiles for the observed data. The green dotted lines represent the 5th and 95th percentiles for the simulated data (after prediction correction). The red asterisk indicates an individual observation falling outside the 90% prediction interval. The shaded areas represent the 90% confidence intervals for the 5th, 50th, and 95th percentiles of the simulated data (*n* = 1000).

A prediction-corrected visual predictive check (pcVPC) based on 1000 simulations of the final model showed that most observed prediction-corrected concentrations fell within the 5th to 95th percentile prediction-corrected intervals and were distributed symmetrically around the prediction-corrected median prediction line, indicating good model fit without apparent bias (Figure 4).

Parameter uncertainty evaluation was conducted using Sampling Importance Resampling analysis. The key PK parameter estimates from the base model, final model, and SIR analysis are summarized in Table 2. The median parameter values from the SIR procedure were consistent with the final parameter estimates. Furthermore, all parameter estimates from the final model fell within the corresponding 2.5–97.5% percentile ranges obtained via SIR. These results support the stability and robustness of the final model against sample variability.

### 3.4. Analysis of the Relationship Between Pharmacodynamic Indices and Clinical Outcomes

The univariable analysis results are summarized in Supplementary Table S3. Higher %fT > MIC showed no statistically significant association with 28-day mortality (Odds Ratio [OR] 0.987, 95% Confidence Interval [CI] 0.968–1.006) or clinical failure (OR 0.987, 95% CI 0.966–1.006). For the binary target of 100%fT > MIC showed no statistically significant association with clinical failure (OR 0.308, 95% CI 0.074–1.038; *p* = 0.080). Each 1 mg/L increase in C_min_ corresponded to a mean prolongation of 0.653 days of mechanical ventilation during the ICU stay (95% CI 0.083 to 1.224; *p* = 0.026). Attaining the aggressive target of 100%fT > 4MIC was significantly associated with a greater reduction in PCT levels compared to non-attainment (Hodges-Lehmann median difference: 2.111, 95% CI 0.144 to 12.795, *p* = 0.027). In the unadjusted bootstrap linear regression, the coefficient for 100%fT > 4MIC was −13.17 (BCa 95% CI: −29.76 to −1.09). After adjusting for SOFA score via propensity score as a covariate, the coefficient was −12.19 (BCa 95% CI: −24.77 to −0.69). The minimal change in the coefficient after adjustment suggested that SOFA score did not substantially confound the observed association.

### 3.5. Model-Based Simulations Results and Optimized Dosing Regimens 

**Figure 5:**
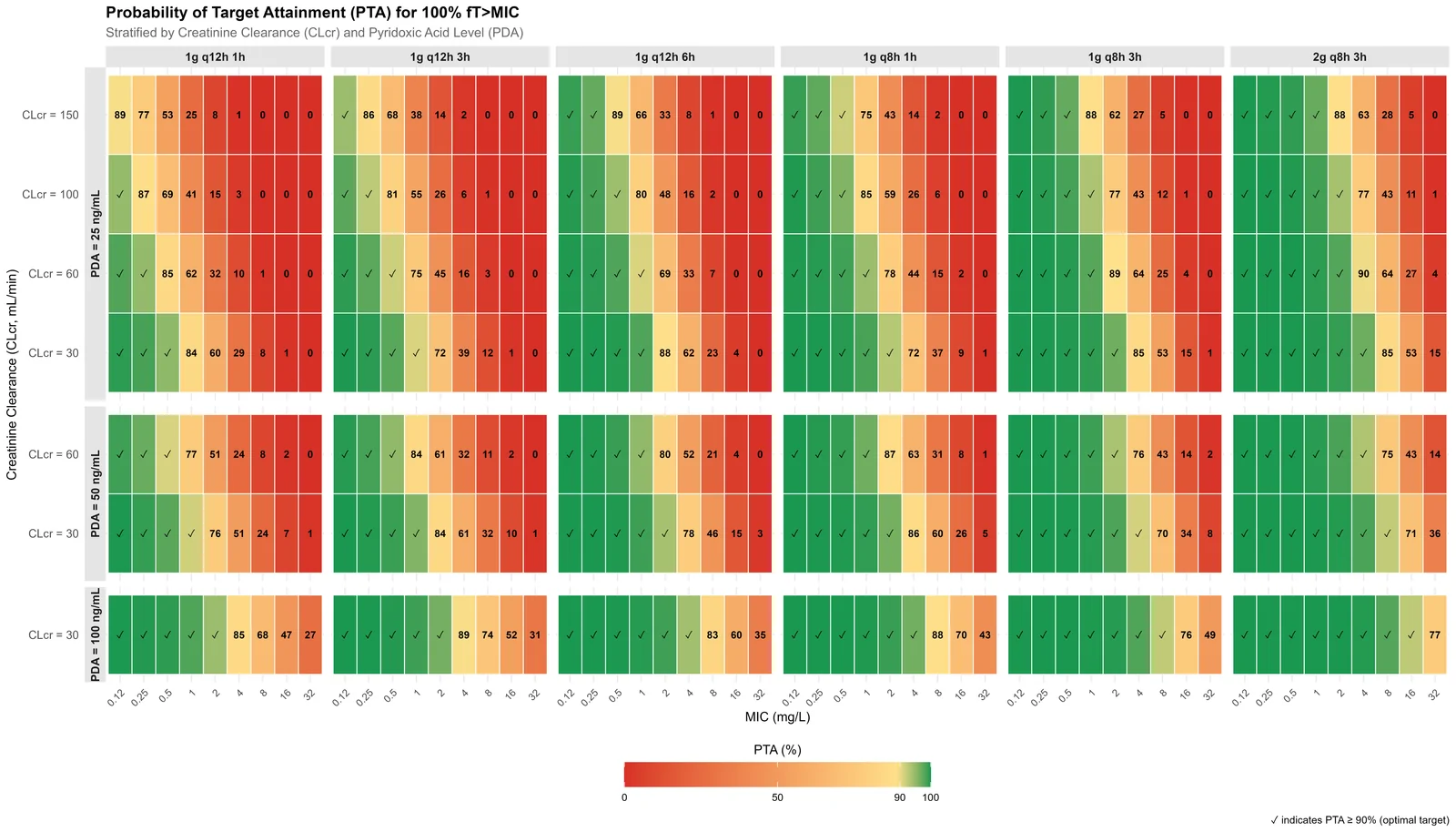
Heat Map of the Probability of Target Attainment (100%fT > MIC) for Various Meropenem Dosing Regimens Against a Range of MICs.

**Figure 6:**
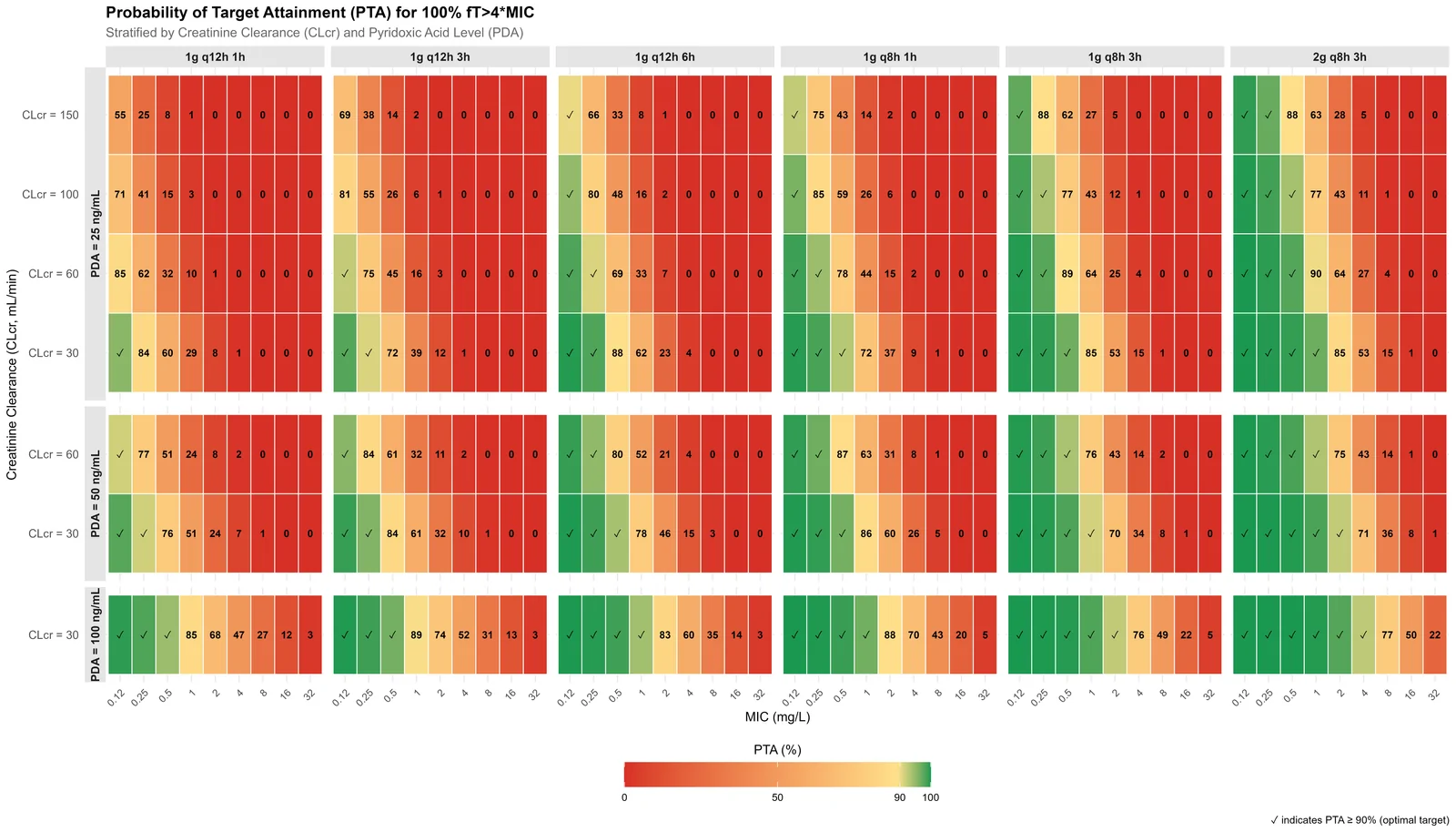
Heat Map of the Probability of Target Attainment (100%fT > 4MIC) for Various Meropenem Dosing Regimens Against a Range of MICs.

The Monte Carlo simulation for PTA demonstrated that the likelihood of achieving the pharmacodynamic target was significantly influenced by CLcr, PDA levels, and MIC. The primary analysis was based on 100%fT > MIC (Figure 5) and 100%fT > 4MIC (Figure 6).

#### 3.5.1. Impact of Covariates on PTA

PDA grouping showed significant pharmacokinetic distinctness. At a fixed CLcr of 30 mL/min, the high PDA group (100 ng/mL) consistently achieved superior PTA compared to the low PDA group (25 ng/mL). For the 1 g q12h 1 h regimen at an MIC of 2 mg/L, PTA in the high PDA group remained robust at 94.48%, whereas it fell to 59.92% in the low PDA group. This suggests that the high PDA level may possess more favorable pharmacokinetic properties for facilitating target attainment.

At a fixed CLcr of 60 mL/min, patients in the high PDA group (50 ng/mL) achieved higher PTA compared to those with low PDA (25 ng/mL). For the 1 g q8h 3 h regimen at MIC 0.5 mg/L, PTA was >90% in the high PDA group but dropped below target in the low PDA group, necessitating dose adjustment.

Renal function was identified as a critical determinant of drug exposure and PTA. At a fixed PDA (50 ng/mL) and dosing regimen, PTA exhibited a marked decline as CLcr increased. In the low CLcr group of 30 mL/min, most simulated regimens maintained high target attainment (PTA > 90%) for MICs ≤ 2 mg/L. Conversely, in groups with higher CLcr of 60 mL/min, accelerated drug elimination resulted in insufficient exposure. For the 1 g q12h 3 h regimen at an MIC of 2 mg/L, PTA dropped from 84.28% in the low CLcr group of 30 mL/min to 60.64% in the higher CLcr group of 60 mL/min), highlighting the increased risk of therapeutic failure in patients with augmented renal function.

#### 3.5.2. Comparison and Optimization of Dosing Regimens

Increasing dosing frequency significantly enhanced PTA. At equivalent daily doses and infusion durations, q8h regimens consistently outperformed q12h regimens. For instance, at CLcr of 60 mL/min and PDA of 25 ng/mL, the 1 g q12h 1 h regimen achieved a PTA > 90% only for MICs ≤ 0.25 mg/L. However, intensifying the frequency to 1 g q8h 1 h extended the effective coverage (Cutoff MIC) to 1 mg/L, with PTA at MIC of 1 mg/L improving from 61.92% (q12h) to 94.50% (q8h).

Extended infusion (3 h) notably improved target attainment at higher MICs, particularly in patients with normal renal function or light renal impairment. Comparing the 1 g q8h regimen (CLcr = 60 mL/min, PDA = 25 ng/mL), extending the infusion from 1 h to 3 h increased the PTA at MIC of 2 mg/L from 77.92% to 89.08%, approaching the 90% therapeutic threshold. For the high-dose 2 g q8h 3 h regimen, extended infusion provided optimal coverage, maintaining >95% PTA even at MICs up to 4 mg/L in the severe renal impairment group.

Doubling the dose (2 g q8h 3 h) yielded the broadest antimicrobial coverage. This was the only strategy capable of achieving substantial coverage at MIC 8 mg/L in the CLcr of 30 mL/min group (PTA of 84.58%), significantly outperforming the standard 1 g dose (PTA of 53.18%).

When applying a stricter PK/PD target of 100%fT > 4MIC (Figure 6), a substantial reduction in PTA was observed across all regimens. Under these stringent conditions, only intensified regimens (e.g., 2 g q8h 3 h or 1 g q8h 3 h in low CLcr settings) could maintain >90% attainment at MIC of 1 mg/L, indicating the necessity for aggressive dosing strategies in scenarios involving refractory infections or high resistance risks.

Collectively, these simulation results support a stratified dosing approach based on both renal function and PDA status. In patients with low PDA (25 ng/mL), even those with moderately reduced renal function may require intensified regimens (1 g q8h 3 h) to achieve target attainment at MIC 0.5 mg/L, whereas in patients with high PDA (50 ng/mL), standard shorter infusions may suffice under the same renal conditions. These findings position PDA as a clinically actionable biomarker for meropenem dose individualization.

## 4. Discussion

In this study, we developed and validated a mechanism-based PopPK model for meropenem in patients with sepsis, and subsequently proposed a precision dosing strategy that integrates both efficacy and safety considerations. The principal novelty of our work is the identification of PDA as a specific endogenous biomarker for renal OAT function. By incorporating PDA into the model, we were able to explained a substantial portion of the IIV in meropenem clearance that conventional markers, such CLcr, failed to capture. Furthermore, our PK/PD analysis revealed a hierarchical relationship between exposure targets and clinical outcomes: the target of 100%fT > 4MIC significantly associated with a significant reduction in PCT levels. Finally, by incorporating a previously established neurotoxicity threshold (C_min_ > 64.2 mg/L) [22], we established a dual-boundary framework for dose optimization, balancing maximal bacterial killing against the risk of drug accumulation.

### 4.1. PDA as a Novel Biomarker for OAT-Mediated Clearance

Consistent with previous studies, our initial model identified CLcr as the dominant covariate for meropenem clearance [10,23,24,25]. highlighting the risk of subtherapeutic exposure in patients with augmented renal clearance [11,26,27,28].

Our study extends these findings by demonstrating that the inclusion of PDA, an endogenous substrate of OAT 1/3, significantly improved the model fit (reduction in OFV by 5.672). This supports the hypothesis that PDA acts as a functional probe for the tubular secretion pathway of meropenem. Biologically, elevated PDA levels reflect impaired OAT function. Since meropenem shares this exact active secretion pathway, a reduction in OAT capacity, mirrored by rising PDA, inevitably leads to decreased meropenem clearance and subsequent drug accumulation.

The data-driven PDA threshold of 26.5 ng/mL estimated by our model is biologically plausible. In healthy populations, baseline PDA levels are approximately 5 ng/mL with wide variability [16]. A possible explanation is that the threshold of 26.5 ng/mL represents the exhaustion of the OAT functional reserve. Below this cutoff, despite a certain degree of functional impairment, the transporters possess sufficient reserve capacity, such that OAT-mediated active secretion does not act as the rate-limiting step for meropenem elimination. However, when PDA exceeds 26.5 ng/mL (a state indicative of severe transporter impairment), the active transport of meropenem becomes saturated. At this juncture, OAT dysfunction becomes the rate-limiting step, leading to the observed linear decline in meropenem clearance.

Consistent with the sensitivity analysis excluding CRRT patients, our findings suggest that in sepsis patients with relatively preserved or homogeneous renal function, traditional CLcr serves as an adequate surrogate for total meropenem clearance. However, in a broader, critically ill population where CRRT introduces substantial heterogeneity, glomerular filtration markers like CLcr may fail to fully capture the true elimination capacity. Under such conditions, the heterogeneity of residual OAT function is markedly amplified, and PDA provides complementary explanatory power for meropenem clearance beyond that of CLcr alone.

Monte Carlo simulations were stratified by PDA levels set at 25, 50, and 100 ng/mL. These values were selected to reflect the empirical data distribution: 25 ng/mL approximates the estimated model threshold (26.5 ng/mL) and serves as the baseline for unimpaired clearance; 50 ng/mL represents the median value (49.6 ng/mL) of the subpopulation exceeding the threshold; and 100 ng/mL represents a severe impairment scenario near the maximum observed concentration (121.3 ng/mL) in the study cohort.

### 4.2. Clinical Implication of Aggressive PK/PD Targets

While traditional conservative targets (e.g., 40–50%fT > MIC) are often insufficient for critically ill patients, achieving aggressive targets (100%fT > 4–5 times MIC) significantly improves clinical cure rates [29].

Our findings align with and extend this body of evidence. We identified a novel association: the attainment of 100%fT > 4MIC was significantly associated with a greater reduction in PCT levels in sepsis. This indicates that patients who maintained drug concentrations above 4 × MIC for the entire dosing interval experienced a significantly more pronounced decrease in this marker of bacterial infection severity. As PCT kinetics reflect the resolution of systemic inflammation, achieving this aggressive exposure likely drives profound bacterial eradication, particularly in patients with severe inflammatory responses. This observation supports the implementation of aggressive targets specifically for patients with high bacterial burden or severe inflammatory responses.

Additionally, we observed a correlation between higher trough concentrations and prolonged days of mechanical ventilation in the ICU. This likely indicates that patients with higher trough concentrations required longer respiratory support, as clinicians may empirically administer higher doses to more critically ill patients requiring prolonged respiratory support.

### 4.3. Balancing Efficacy and Safety: The Neurotoxicity

The pursuit of aggressive PK/PD targets must be tempered by the risk of concentration-dependent toxicity. Although meropenem is generally considered to have a lower neurotoxic potential compared to other beta-lactams such as cefepime, recent pharmacovigilance signals have flagged previously underrecognized risks of seizures [30], and the risk of dose-dependent neurotoxicity cannot be ignored in vulnerable ICU populations [31,32,33].

We adopted the most representative threshold of 64.2 mg/L, as a Cmin exceeding this value has been associated with a 50% probability of neurotoxicity [22]. Our simulation revealed that in patients with severe renal impairment, particularly those with both low CLcr and high PDA (indicating OAT dysfunction), standard high-dose regimens (e.g., 2 g q8h 3 h infusion) pose a significant toxicity risk. For such patients, our model suggests that standard low-dose regimens (e.g., 1 g q8h) are preferable, as they can ensure therapeutic efficacy while minimizing the risk of neurotoxicity.

Notably, no neurotoxicity events (e.g., seizures) were observed in our cohort, likely because the conservative doses administered (0.5 g or 1 g) prevented meropenem concentrations from reaching toxic thresholds. This clinical observation aligns well with our simulation results, which demonstrate that low-dose regimens are preferable for minimizing neurotoxicity risk in patients with severe renal impairment.

### 4.4. A Mechanistic Framework for Precision Dosing

High-risk groups, such as those with renal impairment or elderly, are particularly vulnerable to drug accumulation [34,35]. Conventional dose adjustments based solely on CLcr may fail to identify patients with discordant filtration and secretion functions. Our model-guided approach addresses this gap by integrating PDA to quantify the specific contribution of OAT-mediated secretion to total meropenem clearance.

This mechanistic framework identifies patients with discordant filtration and secretion functions, moving towards functionally informed, rather than merely covariate-guided, precision dosing.

While the measurement of PDA is not yet a routine clinical practice, it offers substantial clinical utility that is complementary to therapeutic drug monitoring (TDM). In institutions where TDM for beta-lactams is unavailable, or during the critical 24-to-48 h window before TDM results are reported, PDA can serve as a sensitive early warning signal.

From a practical standpoint, PDA is quantified by LC-MS/MS, an increasingly available platform in hospital settings. It can be co-measured from routine daily blood samples, imposing no additional burden on patients. For initial implementation, targeted application in high-risk populations, such as patients with severe renal impairment or receiving CRRT, would be a pragmatic strategy. As a proof-of-concept study, we envision that the future availability of commercial assay kits will facilitate its broader clinical adoption.

### 4.5. Limitations

Our study has several limitations that should be acknowledged. First, the relatively small sample size (*n* = 49) and the limited number of clinical events precluded the execution of robust multivariable regression analyses. In critically ill patients, hard clinical endpoints such as 28-day mortality are profoundly driven by baseline disease severity (e.g., SOFA scores). To address this concern specifically for the analysis of PCT reduction while preserving statistical power, we performed bivariate linear regression with bootstrap resampling and BCa confidence intervals, using the propensity score derived from SOFA as the adjusting covariate. The association between 100%fT > 4MIC attainment and PCT reduction remained significant after this adjustment. The consistency of results between unadjusted and adjusted analyses supported the robustness of the finding.

Second, as a single-center study, the generalizability of the established PDA threshold (26.5 ng/mL) and the PopPK model requires external validation in larger, multicenter ICU cohorts. Third, while we identified PDA as a promising biomarker, we did not directly measure OAT expression or genotype polymorphisms (e.g., SLC22A6/SLC22A8 variants). Nevertheless, our findings provide a solid foundation for biomarker-guided precision dosing in septic patients.

A single extreme concentration exceeding 200 mg/L was observed immediately following a 1 h infusion. Given the patient’s adequate renal function, this value is physiologically improbable and indicative of pre-analytical sampling contamination from the intravenous administration line. To maintain data integrity and prevent selection bias, this observation was conservatively retained in the dataset, which did not meaningfully alter the final population pharmacokinetic parameters.

## 5. Conclusions

In conclusion, we have successfully characterized the population pharmacokinetics of meropenem in sepsis and identified PDA as a novel, mechanistically grounded biomarker for renal tubular secretion. By targeting 100%fT > 4MIC to maximal bacterial suppression while limiting C_min_ < 64.2 mg/L to prevent neurotoxicity, we provide a rational, evidence-based foundation for individualizing antibiotic therapy.

## Figures and Tables

**Table 1:** Baseline characteristics of the sepsis patients.

Characteristics	Median (IQR) or *n* (%) ^1^
Demographics and Anthropometry	
Age, yr	74.0 (64.0, 85.0)
Sex, male	18 (36.7%)
Ethnicity, Han	49 (100.0%)
Body-mass index, kg m^−2^	21.5 (18.6, 24.7)
APACHE II score	17.0 (14.0, 21.0)
SOFA score	7.0 (4.0, 10.0)
Social habits	
Current smoking	10 (20.4%)
Ex-smoker > 1 year	13 (26.5%)
Smoking exposure, pack-years	40.0 (22.5, 60.0)
Never-smoker	26 (53.1%)
Current alcohol use	6 (12.2%)
Ex-alcohol > 1 year	6 (12.2%)
Never-drinker	37 (75.5%)
Medical history/Comorbidities	
Chronic lung disease	11 (22.4%)
Hypertension	25 (51.0%)
Diabetes mellitus	19 (38.8%)
Malignancy	6 (12.2%)
Cerebrovascular disease	5 (10.2%)
Surgical history	
Previous surgery	22 (44.9%)
Surgery within 30 days	3 (6.1%)
Laboratory parameters on baseline	
White-cell count, ×10^9^ L^−1^	9.6 (6.6, 14.8)
NT-proBNP, pg mL^−1^	1834.0 (777.0, 4293.0)
Platelet count, ×10^9^ L^−1^	187.0 (99.0, 243.0)
C-reactive protein, mg L^−1^	65.3 (16.6, 125.0)
Procalcitonin, ng mL^−1^	0.6 (0.2, 3.4)
Serum albumin, g L^−1^	31.0 (29.1, 34.8)
Renal-function indices	
Serum creatinine, μmol L^−1^	96.0 (55.0, 166.0)
Creatinine clearance (Cockcroft-Gault) ^2^, mL min^−1^	47.1 (32.8, 80.8)
eGFR (MDRD) ^3^, mL min^−1^ 1.73 m^−2^	62.4 (35.2, 113.5)
Blood urea, mmol L^−1^	12.0 (8.2, 20.7)
24 h urine output, mL	1550.0 (1050.0, 2250.0)
Pyridoxic acid, ng mL^−1^	6.8 (4.3, 17.3)
Renal-status at ICU admission	
AKI	21 (42.9%)
CRRT	7(14.3%)
Non-AKI	21 (42.9%)
Infection details	
Pulmonary	41 (83.7%)
Blood-stream	3 (6.1%)
Polymicrobial infection	30 (61.2%)
Positive microbiological isolate	44 (89.8%)
Outcomes	
All-cause mortality	25 (51.0%)
28-day mortality	19 (38.8%)
In-hospital mortality	24 (49.0%)
Clinical failure	29 (59.2%)
Clinical failure at day 10	27 (55.1%)
ICU length of stay, days	21.0 (14.0, 30.0)
Days on mechanical ventilation, days	4.5 (0.0, 11.0)
ΔCRP (day 5–baseline), mg L^−1^	−25.3 (−72.7, 7.1)
ΔWhite-cell count (day 5–baseline), ×10^9^ L^−1^	−2.2 (−5.1, 0.6)
ΔNeutrophils (day 5–baseline), ×10^9^ L^−1^	−0.6 (−4.7, 1.3)
ΔProcalcitonin (day 5–baseline), ng mL^−1^	−0.4 (−4.2, 0.3)

^1^ Continuous variables are presented as median (interquartile range); categorical variables are presented as number (percentage). ^2^ Creatinine clearance was estimated using the Cockcroft-Gault formula. ^3^ Estimated glomerular filtration rate was calculated using the Modification of Diet in Renal Disease study equation.

**Table 2:** Parameter estimates of the base and final population pharmacokinetic models for meropenem, with Sampling Importance Resampling validation (*n* = 1000).

Parameter	Base Model		Final Model		Sampling Importance Resampling	
	Estimate	95% CI	Estimate	95% CI	Estimate (Medians)	95% CI
Typical value						
CL (L/h)	5.67	4.47, 6.87	7.21	−7.25, 21.68	7.08	5.79, 8.65
Vc (L)	13.4	8.72, 18.08	7.41	3.29, 11.53	7.35	4.99, 9.95
Q (L/h)	5.93	0.36, 11.49	6.45	0.002, 12.90	6.78	3.34, 11.16
Vp (L)	8.94	3.69, 14.19	9.33	3.43, 15.23	9.60	6.92, 12.31
Covariate						
CL-CLcr	-	-	0.29	−0.167, 0.747	0.29	0.09, 0.49
Vc-ALB	-	-	−3.67	−6.12, −1.22	−3.74	−5.28, −2.26
Vc-GROUP	-	-	2.02	0.922, 3.118	2.04	1.28, 2.97
CL-PDA	-	-	−0.042	−0.309, 0.225	−0.043	−0.099, −0.006
IIV	Estimate	Shrinkage%	Estimate	Shrinkage%	Estimate (medians)	95% CI
IIV on CL	50.8%	0.4%	26.4%	2.6%	27.4%	20.7%, 34.3%
IIV on Vc	55.7%	14.1%	22.7%	45.5%	23.2%	10.1%, 32.8%
IIV on Q	123.3%	35.5%	108.6%	29.2%	109.7%	72.4%, 153.3%
Residual Error						
Proportional	0.0516	22%	0.0578	17.5%	0.0601	0.0411, 0.0878
Additive	0.12	22%	0.105	17.5%	0.1065	0.0299, 0.1847

IIV, Inter-individual variation; CL: clearance of meropenem; Vc: volume of distribution of central compartment; Q: clearance of meropenem between central compartment and peripheral compartment; Vp: volume of distribution of peripheral compartment.

## Data Availability

The original contributions presented in this study are included in the article/Supplementary Materials. Further inquiries can be directed to the corresponding authors.

## Supplementary Materials

The following supporting information can be downloaded at: https://www.mdpi.com/article/10.3390/pharmaceutics18091113/s1, Figure S1: Goodness-of-fit diagnostic plots for the final meropenem population pharmacokinetic model; Figure S2: Stratified visual predictive check (VPC) for the target subgroup with elevated PDA (>26.5 ng/mL); Table S1: Liquid chromatography-tandem mass spectrometry (LC-MS/MS) conditions and assay validation parameters for meropenem and pyridoxic acid; Table S2: Stepwise covariate modeling (SCM) procedure for the standard covariate model; Table S3: Summary of univariable analysis of PK/PD targets and outcomes.
