# Supplementary material for: Pyridoxic Acid as a Novel Endogenous Biomarker for OAT-Mediated Clearance of Meropenem: A Population Pharmacokinetic Modeling and PK/PD-Response Analysis in Patients with Sepsis
Source: Pharmaceutics. 2026 Sep 4;18(9):1113. doi: 10.3390/pharmaceutics18091113 (PMC13610781; doi:10.3390/pharmaceutics18091113)
Supplement: Supplementary file 1 [file pharmaceutics-18-01113-s001.zip › pharmaceutics-4457626-supplementary.pdf]

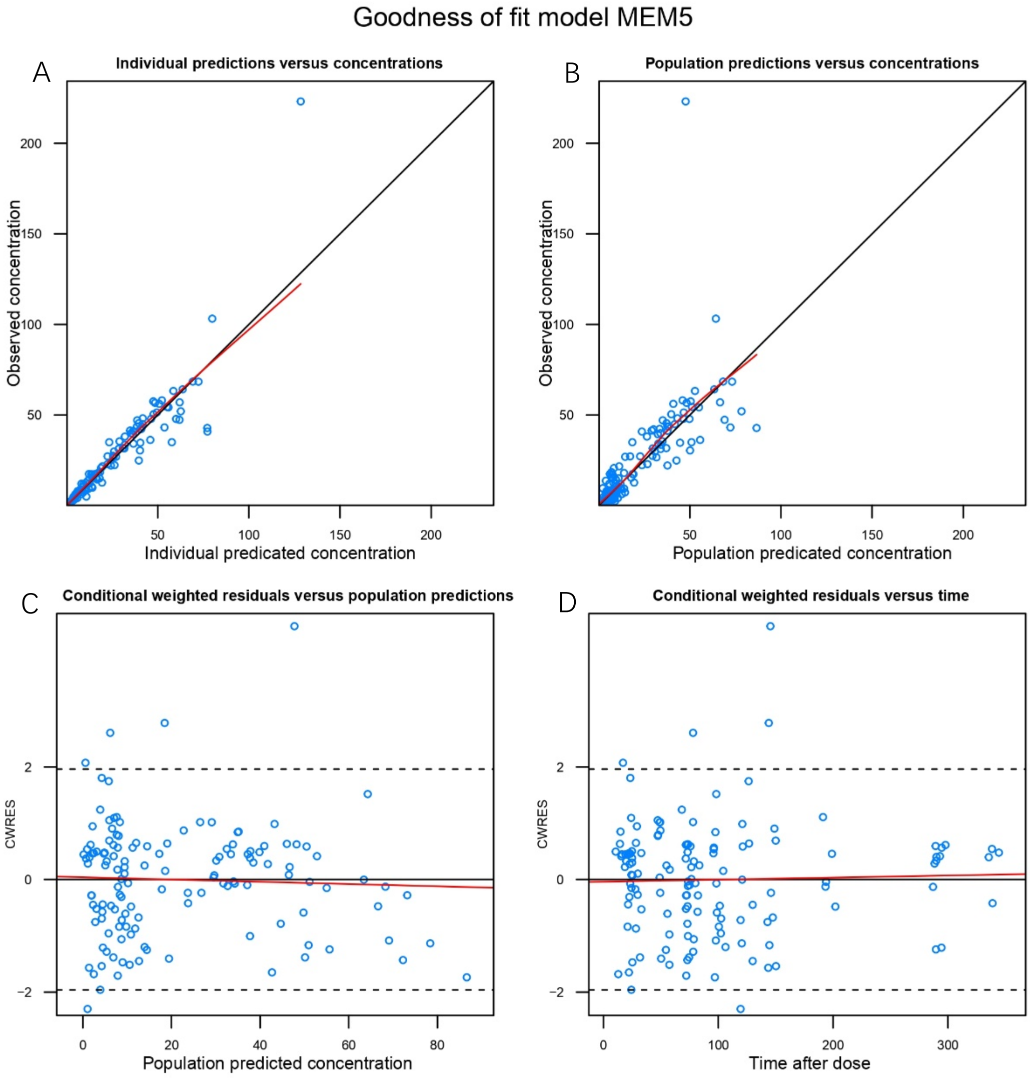

Supplementary Figure S1. Goodness-of-fit diagnostic plots for the final meropenem population pharmacokinetic model. (A) Observed concentrations versus individual population predictions (IPRED). The solid line represents the line of identity (y = x). (B) Observed concentrations versus population predictions (PRED). The solid line represents the line of identity (y = x). (C) Conditional weighted residuals (CWRES) versus population predictions (PRED). The dashed horizontal line represents zero. (D) Conditional weighted residuals (CWRES) versus time after dose. The dashed horizontal line represents zero.

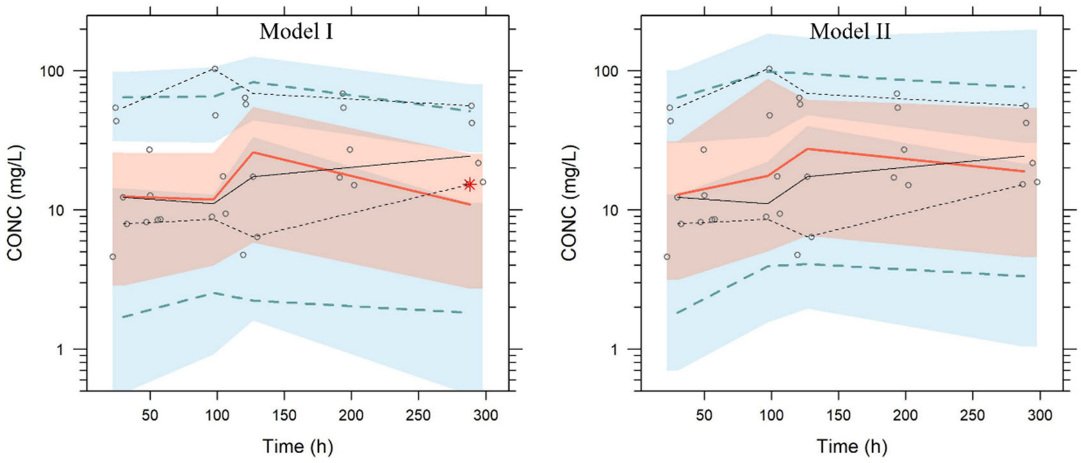

Supplementary Figure S2. Stratified visual predictive check (VPC) for the target subgroup with elevated PDA (> 26.5 ng/mL). A side-by-side comparison between Model 1 (left) and the Model 2 (right). Model 1 exhibits noticeable predictive bias, whereas Model 2 successfully corrects this structural deviation, aligning the empirical observations perfectly within the simulated prediction intervals.

Supplementary Table S1. Liquid chromatography-tandem mass spectrometry (LC-MS/MS) conditions and assay validation parameters for meropenem and pyridoxic acid.

**Parameters**	**Meropenem**	**Pyridoxic Acid**
**Analytical System**	Sciex 4000Q LC-MS/MS	Shimadzu LC-MS 8060NX
**Mobile Phase A**	0.1% formic acid in water	5 mM ammonium acetate with 0.05% formic acid in water
**Mobile Phase B**	0.1% formic acid in acetonitrile	Acetonitrile
**Gradient Elution (Time, %B)**	0.5 min, 1%; 2.5 min, 10%; 4.0 min, 40%; 6.5–8.0 min, 95%; 8.1–11.0 min, 1%	4.5 min, 0%; 5.5 min, 90%; 7.0 min, 90%; 7.5–10.0 min, 0%
**Internal Standard (IS)**	Tolbutamide	Pyridoxic Acid -d3
**MRM Transitions (m/z)**	Analyte: 384.3 → 141.1<br>IS: 271.3 → 172.1	Analyte: 184.1 → 148.15<br>IS: 187.1 → 169.15

**Collision Energy (CE)**	Analyte: 22 V<br>IS: 17 V	Analyte: -21 V<br>IS: -15 V
**Intra- and Inter-day Precision (CV, %)**	QC samples: < 6.97%<br>LLOQ samples: < 9.51%	QC samples: < 14.58%<br>LLOQ samples: < 19.63%
**Intra- and Inter-day Accuracy (RE, %)**	QC samples: < 8.47%<br>LLOQ samples: < 20%	QC samples: < 9.82%<br>LLOQ samples: < 19.84%
**Long-term Stability at -80°C (2 months)**	RE: <±15%	RE: <±15%

Abbreviations: MRM, multiple reaction monitoring; QC, quality control; LLOQ, lower limit of quantification; CV, coefficient of variation; RE, relative error.

Note: Both precision (CV) and accuracy (RE) were within the acceptable limits (i.e., ≤15% for QC samples and ≤20% for LLOQ samples), in accordance with the US Food and Drug Administration (FDA) "Bioanalytical Method Validation - Guidance for Industry" requirements.

Supplementary Table S2. Stepwise Covariate Modeling (SCM) Procedure for the Standard Covariate Model

Step	Model / Covariate Evaluated	OFV	ΔOFV	P-value	Decision
**Base**	**Final Base Model**	**617.96**	-	-	-
**Forward** (*P* < 0.05)					
Step 1	Base + CLcr (Cockcroft-Gault) on CL	607.20	-10.76	0.001	Retained
Step 2	Step 1 + PLT on V_c	601.20	-5.99	0.014	Retained
Step 3	Step 2 + ALB on V_c	595.76	-5.44	0.019	Retained
Step 4	Step 3 + GROUP on V_c	578.15	-17.61	<0.001	Retained
Step 5	Step 4 + 24-hour urine output on CL	573.25	-4.90	0.026	Retained
Step 6	Step 5 + PDA on CL	569.03	-4.22	0.039	Retained
**Full**	**Full Covariate Model**	**569.03**	-	-	**Proceed to backward**
**Backward** (*P* < 0.01)					
Step 7	Full Model - PDA on CL	573.25	+4.22	0.039	Eliminated

Step 8	Step 7 - PLT on V_c	576.42	+3.16	0.075	Eliminated
Step 9	Step 8 - 24-hour urine output on CL	580.97	+4.56	0.032	Eliminated
Step 10	Step 9 - CLcr (Cockcroft-Gault) on CL	590.66	+9.69	0.001	Kept in model
Step 11	Step 9 - ALB on V_c	608.19	+27.22	<0.001	Kept in model
Step 12	Step 9 - GROUP on V_c	606.90	+25.93	<0.001	Kept in model
**Final**	**Standard Covariate Model**	**580.97**	**-**	**-**	**Finalized**

V

Supplementary Table S3. Summary of univariable analysis of PK/PD targets and outcomes

Outcome	Predictor	Odds Ratio (OR) Beta/HL/ ρ	95% CI	*P* value
All cause mortality	%fT>MIC	0.989	0.969-1.007	0.232
	C_{min}	1.015	0.918-1.13	0.773
	C_{max}	1.011	0.978-1.047	0.515
	%fT>4MIC	0.993	0.979-1.006	0.292

	50%fT>MIC	0.559	0.144-2.006	0.379
	50%fT>4MIC	0.764	0.239-2.394	0.644
	100%fT>MIC	0.618	0.182-2.012	0.427
	100%fT>4MIC	0.544	0.16-1.775	0.317
28-day mortality	%fT>MIC	0.987	0.968-1.006	0.173
	C_{min}	0.924	0.791-1.034	0.232
	C_{max}	1.032	0.997-1.073	0.093
	%fT>4MIC	0.992	0.978-1.005	0.224
	50%fT>MIC	0.659	0.18-2.442	0.525
	50%fT>4MIC	0.643	0.197-2.076	0.459
	100%fT>MIC	0.589	0.175-1.97	0.388
	100%fT>4MIC	0.536	0.142-1.82	0.330
In-hospital mortality	%fT>MIC	0.989	0.969-1.007	0.232
	C_{min}	1.015	0.918-1.13	0.773
	C_{max}	1.011	0.978-1.047	0.515
	%fT>4MIC	0.993	0.979-1.006	0.292
	50%fT>MIC	0.559	0.144-2.006	0.379

	50%fT>4MIC	0.764	0.239-2.394	0.644
	100%fT>MIC	0.618	0.182-2.012	0.427
	100%fT>4MIC	0.544	0.16-1.775	0.317
Clinical failure	%fT>MIC	0.987	0.966-1.006	0.207
	C_{min}	0.941	0.836-1.041	0.258
	C_{max}	1.034	0.998-1.078	0.082
	%fT>4MIC	0.99	0.975-1.003	0.138
	50%fT>MIC	0.556	0.131-2.052	0.393
	50%fT>4MIC	0.459	0.131-1.489	0.205
	100%fT>MIC	0.308	0.074-1.083	0.080
	100%fT>4MIC	0.318	0.09-1.059	0.066
Clinical failure at day 10	%fT>MIC	0.987	0.966-1.006	0.184
	C_{min}	0.928	0.816-1.029	0.191
	C_{max}	1.027	0.993-1.068	0.140
	%fT>4MIC	0.989	0.975-1.002	0.116
	50%fT>MIC	0.444	0.105-1.635	0.238
	50%fT>4MIC	0.503	0.15-1.596	0.250

	100%fT>MIC	0.368	0.098-1.24	0.118
	100%fT>4MIC	0.42	0.122-1.378	0.157
PCT Reduction	%fT>MIC[1]	-0.173	-0.433-0.114	0.235
	C_{min}[1]	-0.386	-0.602- -0.118	**0.006**
	C_{max}[1]	0.266	-0.017-0.509	0.065
	%fT>4MIC[1]	-0.226	-0.477-0.059	0.119
	50%fT>MIC[2]	0.728	-0.470-7.047	0.257
	50%fT>4MIC[2]	0.624	-0.677-4.260	0.334
	100%fT>MIC[2]	0.810	-0.360-7.850	0.215
	100%fT>4MIC[2]	2.111	0.144-12.795	**0.027**
WBC Improvement	%fT>MIC	-0.023	-0.063-0.017	0.254
	C_{min}	-0.083	-0.301-0.135	0.449
	C_{max}	-0.061	-0.132-0.01	0.093
	%fT>4MIC	-0.01	-0.039-0.019	0.499
	50%fT>MIC	-1.463	-4.276-1.35	0.301
	50%fT>4MIC	-0.706	-3.254-1.841	0.580
	100%fT>MIC	-1.242	-3.856-1.372	0.344

	100%fT>4MIC	0.104	-2.535-2.743	0.937
NEU Improvement	%fT>MIC	-0.018	-0.056-0.021	0.360
	C_{min}	-0.052	-0.26-0.156	0.617
	C_{max}	-0.046	-0.115-0.022	0.178
	%fT>4MIC	-0.01	-0.037-0.018	0.492
	50%fT>MIC	-1	-3.69-1.691	0.459
	50%fT>4MIC	-0.598	-3.023-1.827	0.622
	100%fT>MIC	-0.829	-3.328-1.67	0.508
	100%fT>4MIC	-0.54	-3.046-1.965	0.667
ICU length of stay	%fT>MIC	-0.034	-0.166-0.098	0.605
	C_{min}	0.282	-0.425-0.988	0.427
	C_{max}	-0.079	-0.315-0.158	0.507
	%fT>4MIC	0.001	-0.093-0.096	0.975
	50%fT>MIC	-3.827	-12.987-5.334	0.405
	50%fT>4MIC	-1.241	-9.524-7.041	0.764
	100%fT>MIC	0.316	-8.243-8.876	0.941
	100%fT>4MIC	-1.127	-9.681-7.427	0.792

days of mechanical ventilation during ICU stay	%fT>MIC	-0.022	-0.134-0.09	0.697
	C_{min}	0.653	0.083-1.224	**0.026**
	C_{max}	0.046	-0.155-0.247	0.645
	%fT>4MIC	0.007	-0.074-0.087	0.870
	50%fT>MIC	-0.944	-8.758-6.869	0.809
	50%fT>4MIC	1.024	-5.993-8.041	0.770
	100%fT>MIC	-0.432	-7.683-6.819	0.905
	100%fT>4MIC	3.224	-3.966-10.414	0.372

[1] Evaluated using Spearman's rank correlation; the effect size is represented by Spearman's correlation coefficient (ρ);

[2] Evaluated using the Mann-Whitney U test; the effect size is expressed as the Hodges-Lehmann estimate (median difference) between groups.
